# Investigating the Association between Catechol-O-Methyltransferase Gene Activity and Pain Perception in South African Patients with Different Temporomandibular Disorders Diagnoses

**DOI:** 10.3390/biomedicines12102331

**Published:** 2024-10-14

**Authors:** Mark Keith Meyer, Enas Ismail, Manogari Chetty

**Affiliations:** 1 Department of Craniofacial Biology, Pathology & Radiology, Faculty of Dentistry, University of the Western Cape, Tygerberg Hospital, Cape Town 7505, South Africa; 2 Physics Department, Faculty of Science (Girl’s Branch), Al Azhar University, Nasr City, Cairo 11884, Egypt

**Keywords:** temporomandibular disorders, Catechol-O-Methyltransferase, single-nucleotide polymorphisms

## Abstract

**Background:** Temporomandibular disorders (TMD) affect a significant portion of the population, with profound psychological, behavioral, and social repercussions. Recent investigations have explored the genetic basis underlying pain perception in individuals with TMD, aiming to elucidate the role of specific genetic factors in modulating the condition. Notably, genetic variations have been implicated in the pathogenesis of TMD, particularly genes involved in pain perception pathways. One of the primary candidates is the Catechol-O-Methyltransferase (COMT) gene, which plays a crucial role in the catecholaminergic system and has been associated with the regulation of nociceptive processes. This study seeks to investigate the correlation between COMT gene activity and pain perception among South African patients diagnosed with varying forms of TMD. **Methodology:** In this study, a total of 196 participants were enrolled, comprising 97 patients diagnosed with TMD and 99 control participants. The control group was meticulously matched with the TMD group for age, gender, and ethnicity. Data collection involved clinical and radiological investigations, and saliva sampling. The English version of the Diagnostic Criteria for Temporomandibular Disorders (DC/TMD) Axis I was utilized to evaluate all TMD participants, focusing on standard diagnostic measures based on clinical signs and symptoms of TMD, which primarily describe common physical manifestations of the disorder. Genomic DNA was extracted from saliva samples, enabling the analysis of single-nucleotide polymorphisms (SNPs) in the COMT gene, specifically targeting polymorphisms rs165774, rs9332377, rs6269, rs4646310, rs165656, and rs4680. **Results:** The current study demonstrated a pronounced gender disparity, with 80.41% of the participants being female and 19.59% male, suggesting that women in South Africa either exhibit a higher susceptibility to TMD or are more likely to seek treatment for the condition compared to men. The highest prevalence of TMD was observed in the white population (58.76%). Additionally, over 65% of TMD patients were diagnosed with at least two Axis I diagnoses, a figure that increased to 89% for those diagnosed with three Axis I diagnoses. The findings further indicated significant associations between several single-nucleotide polymorphisms (SNPs) in the Catechol-O-Methyltransferase (COMT) gene—specifically rs165656, rs9332377, rs4646310, rs6269, and rs165774—and both TMD and TMD-related pain. Myofascial pain with referral and myalgia showed a strong association with the COMT SNPs rs9332377 and rs4646310. Furthermore, COMT SNP rs4646310 was also associated with disability related to TMD. **Conclusions:** This study substantiates the hypothesis that pain is prevalent in a considerable proportion of patients affected by TMD. Furthermore, the findings reveal a significant association between COMT gene activity and pain perception in South African patients diagnosed with TMD.

## 1. Introduction

Temporomandibular disorders (TMD) is a comprehensive term that includes disorders of the temporomandibular joint (TMJ), masticatory muscles, and other musculoskeletal structures associated with the head and neck apparatus [1,2,3,4]. The most common complaint of TMD is pain, which can be associated with either limited mouth-opening and/or TMJ sounds [5,6]. The TMD pain may be of acute onset and self-limiting, and in other situations, it can be persistent [1]. This persistent chronic TMD pain leads to physical, behavioral, psychological, and psychosocial symptoms. Patients may also suffer from chronic pain disorders that affect other areas of the body [5]. This may lead to higher healthcare costs and productivity losses. It is reported that the pain associated with TMD is a substantial challenge for some patients and severely influences all aspects of their daily lives [6]. Research into the role of genetic factors in the etiology of temporomandibular disorders (TMD) has gained traction over the past decade [7]. Several genes implicated in the development of TMD have been identified, particularly those involved in pain perception [8].

The course of chronic pain can be significantly influenced by the single-nucleotide polymorphisms (SNPs) in a gene, specifically target genes associated with the catecholaminergic system, like the Catechol-O-Methyltransferase (COMT) gene, which are linked to the regulation of the nociceptive process [9]. SNPs are recognized as the most prevalent form of genetic variation, which can subsequently impact gene function. A few variants of the COMT gene—rs4646310, rs165656, rs9332377, rs165774, and rs6269—were associated with a high risk for TMD pain. The data suggests that genetic variations in the COMT gene may play a role in the development of TMD pain. SNPs are characterized by a variation in a single nucleotide within the DNA sequence of a gene, where one nucleotide is substituted for another. For example, an SNP may involve the replacement of cytosine (C) with thymine (T). This replacement may occur within exons, introns, or regulatory regions. Gene expression can be changed by interactions between genes and the environment, which can increase or decrease a person’s risk of developing a chronic pain condition. The COMT gene is located on chromosome 22 and codes for a key encoded enzyme that plays a vital role in the catecholamine metabolism, such as dopamine, with a direct relation to pain perception [9,10]. Different genetic parameters affect COMT’s enzymatic activity [11,12]. Numerous SNPs in the COMT gene are associated with TMD [13,14]. The COMT gene plays a crucial role in inactivating various catechol substrates, and in Western populations, polymorphisms in this gene have been significantly associated with an increased risk of developing TMD [15]. Although forty different SNPs have been investigated previously, only a small number of COMT gene variants—rs4646310, rs165656, rs9332377, rs165774, and rs6269—were associated with TMD pain [14,16]. A report of studies on white Americans confirmed the correlation between three COMT genes and high, average, and low pain sensitivity. These included SNPs rs4680, rs4633, and rs4818 [10,17].

The purpose of our study is to investigate the correlation between pain perception in patients with TMD and the association with various COMT gene SNPs in the South African population. The study may assist in the further technological development of a means to identify particular genetic biomarkers that are significant in a patient with TMD who is predisposed to experiencing pain.

## 2. Materials and Methods

### 2.1. Study Participants

This study was conducted during the COVID-19 lockdown period of 2020–2022. Hence, participating patients were from the private sector. The participants were examined and diagnosed at a private practice based in Cape Town, South Africa. They were subsequently divided into White and Non-White ethnic groups. Based on the communication and guidance from the editor of the *South African Medical Journal* and the ethics office at UWC, the terms ‘Black African’, ‘Coloured’, ‘White’, and ‘Indian’ were used.

Only TMD patients older than 18 years old with a confirmed diagnosis, as well as individuals who required dentoalveolar surgery, were included in the study. A total of 196 participants were enrolled; 97 participants presented with a confirmed diagnosis of TMD. Individuals who required dento-alveolar surgery were also included in the study. The remaining 99 participants served as a control group and were age-, gender-, and ethnicity-matched. These patients were randomly assigned over the period of the study. This study employed a set of exclusion criteria to ensure participant homogeneity and minimize confusing factors. The exclusion criteria included the following:

- Maxillofacial trauma or surgery: A detailed clinical history, supplemented by appropriate clinical and radiographic examinations, was used to identify individuals with a history of trauma or surgery in the maxillofacial region. This exclusion aimed to isolate the effects of TMD on pain perception from the potential contributions of prior injuries or surgical procedures.
- Rheumatic conditions: Participants with a confirmed diagnosis of fibromyalgia [18], systemic lupus erythematosus [19], rheumatoid arthritis [20,21], psoriatic arthritis [22], and ankylosing spondylitis [23] were excluded. These chronic inflammatory conditions can independently impact pain perception, potentially confounding the study’s investigation of the relationship between COMT gene activity and pain in TMD patients.

A flow diagram illustrating the stages of the study is represented below.
**Inclusion criteria:** Volunteers of both sexes, 18 years and above.**(*n* = 196)****Exclusion criteria:** History of trauma and/or surgery in the maxillo-facial region; diagnosis of fibromyalgia; systemic lupus erythematosus; rheumatoid arthritis or other forms of systemic joint disease.**Stage I: Clinical diagnosis of TMD** - DC/TMD—Axis I will be applied, and nine groups formed: 0—the absence of TMD; 1—myalgia; 2—myofascial pain with referral; 3—arthralgia; 4—Disk Displacement with Reduction (DDwR); 5—Disk Displacement with Reduction with Intermittent Locking (DDwR-IL); 6—Disk Displacement without Reduction with Limited Opening (DDwoR-LO); 7—Disk Displacement without Reduction without Limited opening (DDwoR-nLO); 8—Degenerative Joint Disease (DJD); 9—subluxation - DC/TMD—Axis II: The graded chronic pain scale, jaw functional limitation scale, patient health questionnaire, general anxiety disorder questionnaire, oral behavior checklist, and patient health questionnaire.**Stage II: Genotyping** Genomic DNA were obtained from saliva samples from all participants.**COMT (Catechol-O-Methyltransferase)** rs165774, rs6269, rs9332377, rs4646310, rs165656, rs4680. 

### 2.2. Ethics

Ethics clearance was obtained from the Faculty of Dentistry, University of Western Cape, Cape Town, South Africa (Ethics no BM 20/4/27). Information sheets and consent were available in three languages: English, Xhosa, and Afrikaans. Voluntary informed consent was obtained from all participants. Participant anonymity was maintained at all times and all data was secured with a password. An updated antivirus program and a unique password safeguard were installed on the author’s personal computer. No information was given to third parties. The study was minimal- to low-risk. Genetic and genomic data obtained from the saliva samples were only used for investigations pertinent to this project and destroyed after the completion of the research.

### 2.3. Saliva Collection Technique

To investigate six particular SNPs in the COMT gene, high-quality, accurate genomic DNA was isolated from saliva. Saliva samples were collected using barcoded Oragene^®^ DGR-400 DNA collection devices, manufactured by DNA Genotek Inc., Ottawa, ON, Canada.

### 2.4. DNA Isolation from Saliva

Accession numbers (1408PCR001 to 1408PCR196) were assigned to the submitted samples and used as laboratory identifiers. Genomic DNA was isolated from saliva samples 1408PCR001–1408PCR100 on the QIAcube device using a QIAamp DNA Blood Mini QIAcube Kit (QIAGEN cat. no. 51126) following the manufacturer’s instructions. Genomic DNA from samples 1408PCR101–1408PCR196 was isolated using the MagMax DNA Multi-Sample Ultra 2.0 Kit (Thermo Fischer cat. no. A36570, Waltham, MA, USA) on the KingFisher Flex following the manufacturer’s instructions. Purity and yield for all genomic DNA was measured by absorbance at A260 and A280 using the Thermo Scientific NanoDrop-8000 Spectrophotometer. All genomic DNA samples were stored at −20 °C until analysis.

### 2.5. Genotyping

Six COMT SNPs were identified using the TaqMan Genotyping Assays (Thermo Fisher cat. no. 4351379). Reactions of 5 µL containing 1X TaqMan^®^ GT Master Mix, 1X SNP genotyping assay mix and 2 µL gDNA were performed in MicroAmp^®^ Optical 384-well Reaction Plates on the QuantStudi-oTM 12K Flex Real-Time PCR System with the following cycling parameters: 95 °C for 10 min, followed by 50 cycles of 92 °C for 15 s, 60 °C for 90 s, and a final 30 s at 60 °C. Genotype calls were assessed using the Thermo Fisher Cloud Connect Platform genotyping application. Genomic DNA extractions from samples in the 3rd and 4th batches (1408PCR101–1408PCR196) were performed using the MagMax DNA Multi-Sample Ultra 2.0 Kit on the KingFisher instrument instead of the QIAamp DNA Blood Mini QIAcube Kit, as specified in the ASP. The KingFisher allows for up to 96 samples per run.

### 2.6. Statistical Analysis

All analyses were executed using R statistical software (https://www.r-project.org/, accessed on 14 September 2024), except for the “Age” variable, which was recorded as a continuous [24]. The processed data comprised only categorical variables. Therefore, all investigations of association were carried out with chi-square or Fisher’s exact test. The Age variable was positively skewed and the Shapiro–Wilk test confirmed that it was not normally distributed (*p*-value ≈ 0.0000) [25]. Consequently, the test of association for Age was implemented using the Mann–Witney non-parametric approach [26,27]. To minimize the false discovery rate due to the use of multiple tests, the Benjamini–Hochberg (1995) [26] correction was used to adjust the *p*-values. Hence, all the *p*-values reported subsequently are adjusted estimates. Finally, the conventional 5% *p*-value threshold was adopted to determine the significance of the evidence present in the analyzed data about each of the explored hypotheses.

## 3. Results and Discussion

### 3.1. TaqMan Genotyping Assay

TaqMan genotyping assays were used to determine the genotype of the six COMT gene SNPs [28]. The assay was used across the 196 investigated samples and a summary of the SNPs used for genotyping is presented in Table 1. The genotype distributions of the six COMT SNPs are summarized in Table 2. It is important to report that PCR amplification was carried out for every SNP analysis using the TaqMan PCR universal master mix and sequence-specific DNA primers on Applied Biosystems (ABI) 7500 Real-Time PCR machine. TaqMan DNA probes with fluorescent reporter dyes at their 5′ ends and non-fluorescent quenchers at their 3′ ends were used to identify each allele. Reverse transcriptase exonuclease activity cleaved the probes as an extension of the SNP primers into the region containing the bound probes, separating the 5′ fluorescent tags from the 3′ non-fluorescent quenchers and producing a fluorescence signal that the ABI 7500 Real-Time PCR instrument detected.

As shown in Table 1, several SNPs within the Catechol-O-Methyltransferase (COMT) gene were listed using their unique rs-ID number [29]. These SNPs represent specific locations in the COMT gene where variations in the DNA sequence occur. The SNPs are important when studying genetic predispositions to various conditions, including pain-related-TMD [30,31,32]. The table shows that SNP-rs165774 (C___2255325_10) involves a substitution of adenine (A) with guanine (G) in the DNA sequence, which may influence the regulation or function of the COMT enzyme and potentially impact pain perception [32]. SNP- rs6269 (C___2538746_1_) shows a guanine (G) to adenine (A) substitution. The polymorphisms in this region of the COMT gene may affect the enzyme’s activity, leading to variations in an individual’s susceptibility to chronic pain. rs4646310 (C__11804672_10) represented a substitution between adenine (A) and guanine (G). Furthermore, the SNP-rs9332377 (C__29614343_10) revealed the substitution of a cytosine (C) with thymine (T). The variations at this site may be attributed to COMT activity and pain sensitivity. This could be a promising marker for the genetic basis of further TMD studies. rs165656 (C___2539305_30) shows a guanine (G)-to-cytosine (C) substitution, which may affect the enzyme’s ability to degrade catecholamines. This may promote pain thresholds and TMD development. Finally, SNP-rs4680 (C__25746809_50) is commonly known as the most well-studied SNP in the COMT gene. It involves a substitution between adenine (A) and guanine (G). This SNP is commonly referred to as the Val158Met polymorphism, where it has been extensively studied in relation to TMD and other pain-related disorders [33,34]. Hence, understanding the role of these COMT SNPs is essential and helps to reveal the genetic factors affecting TMD and pain perception.

**Table 1:** COMT SNPs, genotyped using TaqMan Genotyping assays.

Assay ID	dbSNP	Context Sequence [Allele 1 (VIC)/Allele 2 (FAM)]
C___2255325_10	rs165774	AAACTGGACACTGCTGTTAGCAGCC[A/G]GACTAGGAGCACGAGGGGCACAGCC
C___2538746_1_	rs6269	GCATTTCTGAACCTTGCCCCTCTGC[G/A]AACACAAGGGGGCGATGGTGGCACT
C__11804672_10	rs4646310	TTCTTCAGGGGCTCCAGGAGGACGA[A/G]TGTGTATCCTCCCATTGCTCTGTGC
C__29614343_10	rs9332377	AACCCCTCTCCTTGGGTGCCTCTCC[C/T]TCATAGGCCTGAGTTCCTGGCACTG
C___2539305_30	rs165656	CAGTGCCAGGGTGGGTACAGATTCC[G/C]GCCCGGTGCATGGGCACAGGTCTGC
C__25746809_50	rs4680	CCAGCGGATGGTGGATTTCGCTGGC[A/G]TGAAGGACAAGGTGTGCATGCCTGA

### 3.2. Hardy–Weinberg Equilibrium Analysis

The Hardy–Weinberg Equilibrium (HWE) was applied to investigate any potential genotyping errors. Errors may be present in the genetic data due to different factors, such as family pedigrees and the mishandling of samples [31,35,36]. The presence of such errors usually leads to statistical analyses, which may lead to false data being discussed [34,37]. The principle of HWE describes the expected distribution of genetic variation in a population under certain assumptions. These assumptions may include random mating, no mutation, no migration, no selection, and a large population size. Table 2 shows the COMT SNPs that passed the HWE. The data show the departure of the COMT SNPs rs4646310 and rs4680, which were present in the control group, from the HWE. This could be an indication of the genotype’s protective feature in preventing risk or delaying the onset of the disease phenotype. The COMT SNP rs6269 HWE presents no significance and was stable from one generation to the next. The COMT SNP rs165774 TMD cohort presented with high significance, indicating an association with TMD. The departure from the HWE of the COMT SNP rs165774 TMD cohort indicates that the TMD genotype has not been “fixed” and is still undergoing evolutionary processes. This suggests that the likelihood of inheriting the TMD genotype increases from one generation to the next. Furthermore, the TMD cohort of the COMT SNP rs165656 HWE was not significant due to the low sample size. The control COMT SNP rs165656 was found in HWE and is thus expected to remain stable across generations. COMT SNP rs9332377 HWE was stable from one generation to the next in both the TMD cohort and the control group. Notably, the COMT SNP rs4680 presented significance in both the control group and the TMD cohort. Although the COMT SNP rs4680 TMD cohort presented with significance, the level of significance was much lower than that of the COMT SNP rs165774 TMD cohort and might disappear or become stronger if a larger population is studied.

**Table 2:** The COMT SNPs that passed the Hardy–Weinberg Equilibrium (HWE).

HWE—COMT SNP	CASES	CONTROLS
Rs165656	0.054	0.366
Rs9332377	0.638	0.432
Rs4646310	0.271	0.008
Rs6269	0.251	0.949
Rs165774	0.000	0.440
Rs4680	0.011	0.027

### 3.3. Demographic Factors: Age, Gender, Ethnicity

Participant demographic factors such as age, gender, and ethnicity are represented in Table 3. The data are distributed between the control group (*n* = 99) and the temporomandibular disorder (TMD) cohort (*n* = 97). Table 3 does not show significant differences between the TMD cohort and control groups in terms of gender, age, or race. Regarding the race factor, the study grouped Coloured, Black African, Asian, and Indian as Non-White. The term “White” in South Africa usually refers to people with mostly European ancestry. For the age factor, it is important to report that the youngest participant examined in both groups was 18 years old, while the oldest participants were 79 years old for the TMD cohort and 91 years old for the control group. The difference in the age range resulted in a negligible difference between the average and the median ages of the two groups. Considering gender, although white females formed the largest group in the sample, the percentage of females was not significantly different between the TMD and the control groups. The key demographic factors were analyzed, with corresponding adjusted *p*-values used to evaluate the statistical significance between the different groups. This Table shows a mean age of 36.39 years for the TMD participants and 37.14 years for the control group. Hence, the standard deviation of the control group was slightly higher compared to the TMD group. The Adj *p*-value for the age comparison between the two tested groups, TMD and the control, is 0.8803. This indicates no statistically significant difference. Since both groups have similar average ages and no statistically significant differences, the age factor has a minimum potential effect in this study. The data represented also show the absence of statistically significant differences between the racial distributions, with an adjusted *p*-value of 0.8487. This demonstrates that any pain related to TMD is not based on racial differences. Regarding the gender demographic factor, there was a higher percentage of female participation in both groups: the TMD and the control group. The gender distribution revealed that female participants were 80.4% and 74.7% of the TMD and control groups, respectively, while the male participants comprised 19.59%, and 25.25% of the TMD and the control group, respectively. This is consistent with the reports suggesting a higher prevalence of TMD in females [38]. Disputing the participation % between the females and males, the adj *p*-value was 0.8487, indicating no significant difference based on gender distribution. In conclusion, the demographic data shows the absence of any statistically significant differences between the TMD and control groups. Therefore, the data revealed insignificant evidence for a relation between COMT gene activity and pain perception in South African patients with different demographic characteristics.

### 3.4. Axis I Diagnostic Groups

The Axis I Diagnostic Groups are reported in Table 4. The data revealed no pain in the control group, while the TMD group showed pain in nine subgroups including Myalgia, Myofascial Pain (MFP) with Referral, Arthralgia, Disc Displacement with Reduction (DDwR), Disc Displacement with Reduction and Intermittent Locking (DDwR-IL), Disc Displacement without Reduction and without Limited Opening (DDwR-LO), Disc Displacement without Reduction and without Limited Opening (DDwoR-nLO), Degenerative Joint Disease (DJD), and Subluxation, as shown in Table 4. The data revealed that more than 65% and 89% of the TMD patients were diagnosed with two Axis I diagnoses and three Axis I diagnoses, respectively. Pain was reported for the TMD patients with a maximum *p*-value of 0.0053 in all Axis I diagnostic groups except for subluxation, with a *p*-value of 0.2853. Notably, the subluxation group’s unadjusted *p*-value (*p* = 0.0581) was considered insufficient as it only represents 4.12% of the sample. Hence, further studies are essential for TMD patients who feel subluxation pain. The represented data revealed that Groups 7 and 8 (DDwoR-nLO, and DJD, respectively) showed the most relevant TMD symptoms. Over 40% of the participating patients reported the presence of pain. Muscle involvement, either as Myalgia or Myofascial Pain (MFP), was observed in 66% of the TMD cohort, specifically in Groups 1 and 2. In conclusion, all diagnostic TMD groups in this study experienced a degree of pain.

### 3.5. Distribution of COMT Genotype Frequencies between the TMD and the Control Groups

The genotypic compositions of the COMT SNPs and their associations with the nine diagnostic groups were investigated using data from patients presenting with TMD pain only. Table 5 presents the distribution of COMT genotype frequencies between temporomandibular disorder (TMD) patients and a control group, providing insights into the potential association of specific single-nucleotide polymorphisms (SNPs) with TMD. The odds ratios (OR), confidence intervals (CI), and *p*-values for each genotype within the SNPs were analyzed and are presented within this Table to assess statistical significance.

This Table summarizes the results of both the tests of association and regression analyses. The regression models all included demographic variables such as age, race, and gender. The distributional pattern of the genotypes was similar for both the control and the TMD groups. It is noteworthy, however, that within groups, the genotypes were most evenly distributed for most COMT SNPs except for COMT SNP rs165774. COMP SNPs rs165774 genotype AG (*p* = 0.0225) and rs4680 genotype GG (*P* = 0.0498) presented a significant association with the studied sample. A significant finding was that the COMT SNP rs4646310 AA genotype only appeared in 2% of the TMD cohort and 6% of the control group, compared to 20% and 17% for AG, and 74% and 75% for GG. Notably, while the GG genotype shows the possibility of conveying a higher TMD risk, the AG genotype has a vital protective effect against TMD. This explains the strong interaction relation between these SNPs and the TMD risk. Most of the COMP SNP odds ratios (ORs) were fairly close to 1, suggesting that the genotypic distribution between the case and control sample remained similar throughout. This is also shown by the lack of any statistical significance. It is important to note that the COMT SNP rs165774 AG genotype (OR = 0.489) is less than 1, which may indicate a protective factor against TMD disorder. This contrasts with the COMT SNP rs4680 GG genotype (OR = 1.9091), which is greater than 1, indicating that this might be a risk factor for developing TMD. SNP rs4680 is shown to play a vital role in modulating pain sensitivity. The data show that both rs165774 and rs4680 genotypes are linked to TMD-related risk. The GG genotype in both SNPs is associated with increased TMD pain sensitivity. However, more comprehensive studies of different populations are essential to reveal the exact relation among the different SNPs and their pain correlation.

In conclusion, the data presented on the COMT distribution genotype frequencies highlight the potential effects of the COMT SNPs, particularly rs165774 and rs4680. The data reveals their potential association with the risk of developing TMD in the studied population. The AG genotype of rs165774 appears to have a protective effect, while the GG genotype of rs4680 is linked to an increased risk of TMD [39,40]. The current results align with other reported publications about TMD patients, and the presence of higher pain sensitivity and longer pain chronicity [41].

Notably, other SNPs did not reveal statistically significant associations with TMD. Genetic factors may play a significant role in TMD development; however, other factors should be considered. Hence, further investigations including a larger sample size and additional factors are recommended.

**Table 5:** Distribution of COMT genotype frequencies between the TMD and the control groups.

SNP	TMD (%)	Control (%)	OR	CI	*p*-Value
rs165656					
**CC**	27	22	1.3717	0.7153 to 2.6304	*p* = 0.3415
**CG**	38	44	0.8182	0.4619 to 1.4494	*p* = 0.4915
**GG**	30	32	Ref	Ref	Ref
rs9332377					
**CC**	66	66	Ref	Ref	Ref
**CT**	27	26	1.069	0.5673 to 2.0144	*p* = 0.8365
**TT**	2	4	0.4946	0.08842 to 2.767	*p* = 0.4229
rs4646310					
**AA**	2	6	Ref	Ref	Ref
**AG**	20	17	1.2539	0.6113 to 2.5718	*p* = 0.5371
**GG**	74	75	1.0806	0.5512 to 2.1184	*p* = 0.8214
rs6269					
**AA**	40	42	0.9694	0.5489 to 1.712	*p* = 0.9147
**AG**	40	45	0.8571	0.4863 to 1.5107	*p* = 0.5939
**GG**	16	12	Ref	Ref	Ref
rs165774					
**AA**	16	10	Ref	Ref	Ref
**AG**	25	37	**0.489**	0.2644 to 0.9042	***p* = 0.0225**
**GG**	60	45	1.5285	0.8644 to 2.7025	*p* = 0.1446
rs4680					
**AA**	28	26	Ref	Ref	Ref
**AG**	35	37	0.9302	0.518 to 1.6704	*p* = 0.8085
**GG**	32	33	**1.9091**	1.0005 to 3.6428	***p* = 0.0498**

## 4. Conclusions

This study aimed to explore the association between specific single-nucleotide polymorphisms (SNPs) in the Catechol-O-Methyltransferase (COMT) gene and pain perception in South African patients diagnosed with temporomandibular disorders (TMD). Given the established role of the COMT gene in modulating pain, the study focused on understanding how genetic variations may influence susceptibility to TMD, a condition known for its complex and multifactorial nature. A comprehensive genetic analysis was conducted, examining six key COMT SNPs in both TMD patients and a control group. The demographic distribution between the two groups was carefully balanced, with no significant differences in age, race, or gender, thus ensuring that these variables did not confound the results. The analysis revealed notable findings, particularly regarding rs165774 and rs4680 polymorphisms. The AG genotype of rs165774 was found to be significantly less frequent in the TMD group compared to the control group, suggesting a potential protective role against TMD (*p* = 0.0225). Conversely, the GG genotype of rs4680 showed a significant borderline association with increased TMD risk (*p* = 0.0498), reinforcing the role of COMT in pain perception and TMD susceptibility. These genetic associations align with the reported research about the importance of COMT in TMD patients. In conclusion, this study provides valuable insights into the genetic underpinnings of TMD, highlighting the influence of COMT polymorphisms on pain perception and TMD risk. The findings underscore the potential for genetic screenings to identify individuals at higher risk for TMD, which could lead to more personalized and targeted therapeutic interventions. The clinical significance of the current study relies on possible improvements in treatment decisions based on genetic profiles in a variety of clinical conditions involving pain and neurodegenerative diseases. Future studies with larger and more diverse populations are recommended to confirm these findings in different nations to explore the broader implications of COMT gene variations in pain-related disorders. Additionally, integrating genetic information into clinical practice could enhance the management and treatment outcomes for TMD patients

## Figures and Tables

**Table 3:** The distributions of the participants regarding their demographic factors.

		TMD		CONTROL		Adj. P
**Age**						0.8803
	n	97		99		
	%	49.49		50.51		
	Mean	36.39		37.14		
	SD	14.72		15.12		
		N	%	N	%	
**Race**						0.8487
	NonWhite	40	41.24	46	46.46	
	White	57	58.76	53	53.54	
**Gender**						0.8487
	Male	19	19.59	25	25.25	
	Female	78	80.41	74	74.75	

**Table 4:** Axis I diagnostic groups.

		TMD		CONTROL		Adj. P
Myalgia (Group 1)		N	%	n	%	<0.0001
	No	68	61.86	99	100	
	Yes	29	29.90	0	0.00	
MFP with Referral (Group 2)						<0.0001
	No	60	61.86	99	100	
	Yes	37	37.14	0	0.00	
Arthralgia (Group 3)						<0.0001
	No	76	78.35	99	100	
	Yes	21	21.65	0	0.00	
DDwR (Group 4)						<0.0001
	No	70	72.16	99	100	
	Yes	27	27.84	0	0.00	
DDwR-IL (Group 5)						0.0041
	No	84	86.60	99	100	
	Yes	13	13.40	0	0.00	
DDwR-LO (Group 6)						<0.0001
	No	58	59.79	99	100	
	Yes	39	40.21	0	0.00	
DDwoR-nLO (Group 7)						0.0053
	No	87	89.69	99	100	
	Yes	10	10.31	0	0.00	
DJD (Group 8)						<0.0001
	No	56	57.73	99	100	
	Yes	41	42.27	0	0.00	
Subluxation (Group 9)						0.2853
	No	93	95.88	99	100	
	Yes	4	4.12	0	0.00	

## Data Availability

The data presented in this study is available on request from the corresponding author.
